# Physical Biology in Cancer. 3. The role of cell glycocalyx in vascular transport of circulating tumor cells

**DOI:** 10.1152/ajpcell.00285.2013

**Published:** 2013-10-16

**Authors:** Michael J. Mitchell, Michael R. King

**Affiliations:** Department of Biomedical Engineering, Cornell University, Ithaca, New York

**Keywords:** metastasis, adhesion, drug delivery, microvasculature

## Abstract

Circulating tumor cells (CTCs) in blood are known to adhere to the luminal surface of the microvasculature via receptor-mediated adhesion, which contributes to the spread of cancer metastasis to anatomically distant organs. Such interactions between ligands on CTCs and endothelial cell-bound surface receptors are sensitive to receptor-ligand distances at the nanoscale. The sugar-rich coating expressed on the surface of CTCs and endothelial cells, known as the glycocalyx, serves as a physical structure that can control the spacing and, thus, the availability of such receptor-ligand interactions. The cancer cell glycocalyx can also regulate the ability of therapeutic ligands to bind to CTCs in the bloodstream. Here, we review the role of cell glycocalyx on the adhesion and therapeutic treatment of CTCs in the bloodstream.

metastasis contributes to ∼90% of cancer-related deaths (13, 128), yet many aspects of metastasis remain poorly understood. Cancer cells originating from the primary tumor undergo a sequence of steps to metastasize via the bloodstream to anatomically distant organs, including detachment from the primary tumor, invasion into surrounding tissues, and intravasation into the vascular circulation as circulating tumor cells (CTCs) (14, 132). CTCs can then be transported through the vascular system to the postcapillary venules of distant tissues, undergo adhesive interactions with the microvessel wall, exit the bloodstream in a process known as extravasation, survive in distant tissues, and proliferate to form secondary tumors (28). While primary tumors are generally treatable via radiation, chemotherapy, and/or surgical removal, the systemic nature of metastasis makes the disease difficult to treat (66). A better understanding of the vascular transport of CTCs can reveal key checkpoints for the intervention and treatment of metastasis.

Receptor-ligand interactions play a key role in the adhesion and therapeutic treatment of CTCs in the bloodstream. To adhere to the microvasculature in distant tissues, sialylated carbohydrate ligands expressed on CTCs can bind to selectin receptors on the surface of inflamed endothelial cells (ECs) (19, 28). This adhesion mechanism has been used in recent biomimetic approaches to target CTCs via immobilized E-selectin receptors under physiological flow conditions (66, 97, 98). Such techniques can allow flowing cancer cells to interact with apoptosis-inducing ligands (97, 98), which can bind with receptors on the cancer cell surface to trigger programmed cell death. The ability of CTCs to undergo such receptor-ligand interactions can be dictated by a physical barrier on the surface of cells known as the glycocalyx.

The glycocalyx is a sugar-rich coating that is found on the surface of ECs and tumor cells. The EC glycocalyx serves as a vascular permeability barrier, a mechanotransducer of hemodynamic shear forces to ECs, and a regulator of adhesive interactions between circulating cells and the endothelium (129). Tumor cells can overexpress certain building blocks of the glycocalyx, which can facilitate tumor progression by enhancing angiogenesis, tumor growth, and invasion (121). Given that this layer can approach a thickness of 0.5 μm while receptors are mostly <100 nm in length, the glycocalyx can act to control receptor interactions with their respective ligands (71, 129). Thus the thickness of the glycocalyx can affect CTC adhesion to the endothelium, along with therapeutic ligand delivery to the surface of CTCs.

Here, we discuss a range of potential effects on the vascular transport of CTCs due to the glycocalyx. First, the structure and composition of the glycocalyx, found on ECs and tumor cells, is reviewed. The factors that contribute to EC glycocalyx remodeling and disruption are then described, along with their subsequent effects on the adhesion of circulating cells. We conclude with novel therapeutic strategies for CTCs, the glycocalyx as a barrier for CTC drug delivery, and approaches to disrupt the glycocalyx for efficient therapeutic treatment of CTCs.^1^

## EC Glycocalyx Structure

**Fig. 1.**
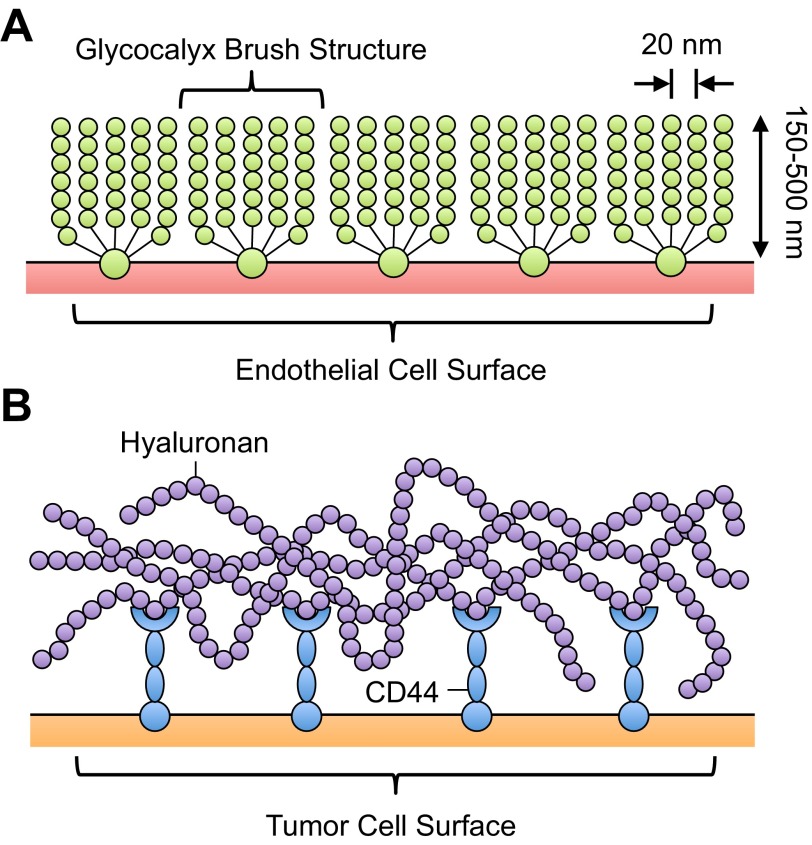
Schematics of the endothelial (*A*) and tumor (*B*) cell glycocalyx (not drawn to scale). *A*: endothelial cell glycocalyx can extend 150–500 nm from the endothelial cell surface, with a typical spacing of 20 nm between glycocalyx components. [Adapted from Weinbaum et al. (130).] *B*: tumor cell glycocalyx consists of similar components but is characterized by greater content of hyaluronan, which is tethered to the tumor cell membrane by CD44.

The structure of the EC glycocalyx is discussed here briefly, as this has been discussed in detail by others (71, 95, 101, 129). The glycocalyx, with an estimated thickness of 150–500 nm, is a thin, gel-like layer of macromolecules on the apical surface of vascular ECs (129) (Fig. 1*A*). Glycocalyx measurements are based on in vivo experimental observations by Vink and Duling (125) using intravital microscopy, electron microscopy studies by van den Berg et al. (124), and others (20, 21, 105). The glycocalyx on the surface of postcapillary venules has been measured using capillary tube hematocrit, defined as the instantaneous volume fraction of postcapillary venules filled with red blood cells (55, 58, 107). Reductions in the perfused capillary volume are indicative of the glycocalyx extending from the EC surface (22, 125). Electron microscopic images by Squire et al. (114) showed that the EC glycocalyx brush structure has a characteristic spacing of 20 nm in all directions (Fig. 1*A*). Computational models (45, 77) and experimental observations (1, 90) have shown that such spacing can act as a “molecular sieve” for plasma proteins and, thus, create differences in plasma protein concentration between tissue and the luminal surface of the endothelium.

The glycocalyx on the EC surface primarily consists of glycosaminoglycans (GAGs), linear heteropolysaccharides with characteristic disaccharide unit repeats (51). GAGs that comprise the EC glycocalyx include heparan sulfate, hyaluronan, and chondroitin sulfate (88). Sulfated GAGs, specifically heparan sulfate and chondroitin sulfate, are linked to EC membrane-bound proteoglycans, which link the glycocalyx to the actin cytoskeleton (114). Proteoglycans are proteins that have specific sites to covalently link sulfated GAGs and consist of transmembrane syndecans, membrane-bound glypicans, and matrix-localized perlecans (104). Hyaluronan does not possess sulfated groups and is not covalently linked to proteoglycans; its interaction with the glycocalyx is mediated by cell surface receptors such as CD44, as well as by chondroitin sulfate chains (40). Other important glycoproteins on the cell surface include adhesion receptors, such as integrins, immunoglobulins, and selectins (112). Under normal physiological conditions, various blood-borne proteins will also incorporate into the EC glycocalyx (47).

## Tumor Cell Glycocalyx Structure

The tumor cell glycocalyx, much like the EC glycocalyx, consists of a variety of proteoglycans and GAGs, in addition to fibrous proteins, such as collagen, which comprise the surrounding extracellular matrix (109). The synthesis of hyaluronan in tumor cells, however, is frequently impaired during malignant transformation and can result in the excess production of hyaluronan (39, 43, 48, 65) (Fig. 1*B*). In nontransformed cells, hyaluronan incorporates into the surrounding cell matrix by forming aggregates with hyaluronan-binding molecules and can regulate cell adhesion, motility, growth, and differentiation. Hyaluronan synthase genes (HAS1, HAS2, and HAS3) encode key enzymes in hyaluronan synthesis, which can regulate the ability to form hyaluronan matrices and determine hyaluronan molecular size (50). In tumor cells, however, expression of hyaluronan synthase genes is often increased, resulting in excess hyaluronan production. Experiments forcing expression of HAS2 and HAS3 resulted in a drastic increase in hyaluronan production and subsequent tumorigenicity of mesothelioma, melanoma, and fibrosarcoma (60, 67, 72). Additionally, transfection of HAS1 into mouse mammary carcinoma mutants rescued hyaluronan matrix production and metastatic potential (49). Expression of the hyaluronan cell surface receptor CD44 has also been shown to be increased in tumor cells (34, 57) (Fig. 1*B*). CD44^+^ cancer cells from head and neck squamous cell carcinoma can possess properties of cancer stem cells, including cancer stem cell renewal and differentiation (96). CD44 variant isoforms are highly expressed in carcinomas of epithelial origin and relate to tumor progression and metastatic potential of some cancers (38, 87, 108). In addition to these components, a variety of cell and matrix adhesion molecules, including integrins and selectin ligands, are embedded in the glycocalyx of tumor cells (33, 115).

The CTC glycocalyx has not been well characterized. Paszek et al. (91) recently developed scanning angle interference microscopy to measure variations in glycocalyx thickness of single epithelial cells on the nanometer scale. This technique could be utilized to characterize the glycocalyx of CTCs. While usually associated with disease progression and poor prognosis, a significant number of CTCs in blood are typically apoptotic (76, 92). This is in part due to anoikis, a form of programmed cell death due to the loss of cell-cell and/or cell-matrix adherence in CTCs (137). This raises the intriguing possibility that viable CTCs that contribute to metastasis, which are typically associated with the epithelial-mesenchymal transition (54, 118, 136), can also retain their glycocalyx and other matrix components. The glycocalyx coating could allow CTCs to evade anoikis or other forms of cell death due to harsh shear stress exposure in the circulation. In particular, clusters of CTCs, known as circulating tumor microemboli, can retain their viability, in part due to retention of glycocalyx, surrounding matrix components, and cell-cell adherence, as well as processes such as the epithelial-mesenchymal transition (24, 44, 54).

## EC Glycocalyx Effects on CTC Adhesion

It is believed that CTCs can leave the bloodstream during hematogenous metastasis in a manner similar to leukocyte extravasation during the inflammatory response. In this process, cells initially tether and roll on the activated endothelium, firmly adhere, and then transmigrate through the blood vessel wall into inflamed tissue (15, 19). The initial tethering and rolling of leukocytes to ECs is mediated by E-, L-, and P-selectin binding to ligands on the microvilli of leukocytes on the surface of ECs (62–64, 80), with firm adhesion mediated by ICAM-1 and β_2_-integrins on the EC and leukocyte surfaces, respectively (46, 113). E-selectin on the EC surface has also been shown to facilitate cancer metastasis in vivo (5, 9). Additionally, E-selectin can induce the rolling and tethering of cancer cells originating from breast (32, 122), colon (11, 122), and prostate (4, 23) under flow.

**Fig. 2.**
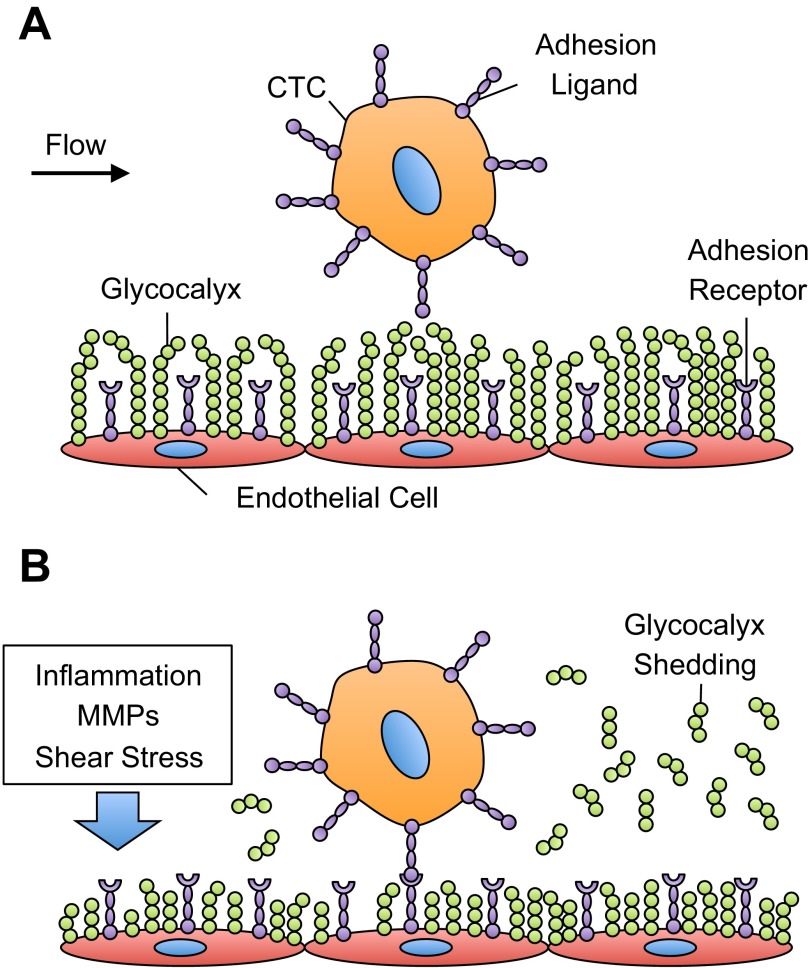
Glycocalyx effects on circulating tumor cell (CTC) adhesion in the microvasculature. *A*: under normal physiological conditions, the endothelial cell glycocalyx thickness is greater than the length of most adhesion receptors and acts as a barrier to cell adhesion. *B*: alterations in shear stress, inflammatory conditions, and matrix metalloproteinase (MMP) exposure can cause shedding and/or remodeling of the glycocalyx, increasing the number of available receptors to bind to adhesion ligands on CTCs.

The EC glycocalyx can control the spacing between E-selectin receptors on the EC surface and selectin ligands on CTCs; however, its effects on CTC adhesion to the blood vessel wall have received less attention. Cell adhesion molecules on the EC surface, such as ICAM-1 and selectins, can range in length from 20 to 40 nm (12, 112). However, the thickness of the glycocalyx can be several hundreds of nanometers and, thus, affects receptor-mediated cell adhesion under physiological flow (126, 129) (Fig. 2*A*). For example, Robert et al. (103) utilized computational and experimental models to measure the effect of the glycocalyx layer on the adhesion of functionalized microbeads to immobilized ICAM-1 under flow. Using hyaluronan as a model glycocalyx, they showed an increase in the frequency of adhesion to ICAM-1 under flow with decreasing concentrations of hyaluronan, along with an increase in the force between the bead and the substrate and a decrease in the bead distance from the surface (103). Multiple studies using leukocytes have shown that decreases in glycocalyx thickness directly correlate with increased cell adhesion (106, 110, 111).

Inflammation, exposure to extracellular proteases, and changes in hemodynamic shear stress can alter glycocalyx molecular composition and thickness (Fig. 2*B*), suggesting that such factors may promote or inhibit CTC-EC adhesion.

## 

### Glycocalyx remodeling during inflammation.

The EC glycocalyx can be dramatically remodeled during inflammation, a process that is also critical to the progression of cancer metastasis (19, 35). In vivo, Henry and Duling (41) found that exposure to the proinflammatory cytokine TNF-α can disrupt and increase the entry of macromolecules into the EC glycocalyx. Mulivor and Lipowsky (86) showed that superperfusion of the chemoattractant formyl-methionyl-leucyl-phenylalanine (fMLP) into rats induced significant shedding of GAGs from the EC surface. Treatment of postcapillary venules with fMLP or heparinase increased exposure and availability of ICAM-1 on the EC surface (85), as measured by perfusion and adhesion of fluorescent microbeads coated with antibodies specific for ICAM-1. In an in vivo model of septic shock, administration of bacterial endotoxins induced a significant increase in circulating glycocalyx degradation products such as heparan sulfate (42). Proinflammatory LDLs have been shown to degrade the EC glycocalyx in postcapillary venules, as measured by increased capillary hematocrit (17). There is also evidence that major cardiopulmonary bypass surgery can cause shedding of heparan sulfate and syndecan-1 from the EC glycocalyx of patients (99). Given the increased thickness of the glycocalyx under normal physiological conditions, it is likely that inflammatory conditions promote CTC adhesion to the endothelium by shedding and disruption of the EC glycocalyx, which increases the availability of EC adhesion molecules to CTCs.

### Matrix metalloproteinase effects on glycocalyx shedding.

Extracellular proteases, such as matrix metalloproteinases (MMPs), can induce glycocalyx shedding and promote cell adhesion in the microvasculature (Fig. 2*B*). MMPs are a family of zinc-dependent enzymes that can regulate the turnover of the glycocalyx and other extracellular matrix components during processes including inflammation, wound healing, and tumor progression (19, 73, 74, 83). In vivo, arterial ECs show increased expression of MMPs in areas of atherosclerotic plaques and lesions (31, 68). MMPs have also been found by Gronski et al. (36) and Endo et al. (26) to directly cleave chondroitin sulfate and proteoglycan syndecan-1, respectively.

ECs have the ability to store, activate, and release MMPs into the surrounding glycocalyx. Utilizing MMP inhibitors, Fitzgerald et al. (29) found that multiple cell signaling pathways can converge and activate MMPs, after PMA treatment, to cleave syndecans from the cell surface. Taraboletti et al. (117) found that human umbilical vein ECs shed microvesicles from the plasma membrane; these microvesicles contain active and proenzyme forms of MMP-2 and MMP-9 (117). Yu and Woessner (138) found evidence that MMP-2 and MMP-9 can bind to heparan sulfate in the glycocalyx. ECs were also found to utilize microvesicles to release tissue inhibitors of metalloproteinases, endogenous inhibitors that can regulate MMP activity (117). Thus, shedding of the glycocalyx can, in part, be regulated by MMPs derived from the endothelium. Given that nearly all tumor cells overexpress MMPs (18), including MMP-2 and MMP-9 as mentioned previously, CTCs may also contribute to MMP-induced glycocalyx shedding.

### Glycocalyx response to fluid shear stress.

The EC glycocalyx is exposed to hemodynamic shear stresses of 4.0–30.0 and 0.5–4.0 dyn/cm^2^ in the arterial and venous circulation, respectively (123). Shear rates can range from ∼900 s^−1^ in arteries to 160 s^−1^ in veins (82). Exposure to such forces can affect the biosynthesis of EC glycocalyx components, activate EC-derived proteases, and disrupt glycocalyx structure and molecular components (2). In vivo studies by Mulivor and Lipowsky (86) showed that induction of ischemia for 60 min led to an increase in glycocalyx thickness on the surface of the postcapillary venules, which then decreased upon reperfusion of the venules. Grimm et al. (34a) showed that exposure to low shear stress (1.0 dyn/cm^2^) inhibited GAG synthesis in cultured ECs, while Arisaka et al. (2) showed that greater shear stresses (>15.0 dyn/cm^2^) stimulated GAG synthesis in ECs. Zeng et al. (139) found that specific components of the EC glycocalyx, such as heparan sulfate, can cluster at EC junctions through the mobility of glypican-1 in lipid rafts. Other components, such as chondroitin sulfate and syndecan, remained immobilized on the EC glycocalyx (139). Koo et al. (59) measured the components of the glycocalyx after human EC exposure to shear stress waveforms characteristic of atherosclerosis-resistant and atherosclerosis-susceptible regions of the arteries. Glycocalyx components increased in expression and were distributed evenly on the EC surface after exposure to atherosclerosis-resistant waveforms but were irregularly distributed and decreased in expression upon exposure to atherosclerosis-susceptible waveforms (59).

In the tumor microenvironment, blood vessels are characterized as disorganized, tortuous, leaky, and dilated (52). While normal vessels typically branch via bifurcations with even branch diameters, the tumor vasculature can exhibit trifurcations and uneven branch diameters (52), all of which can dramatically alter the local shear stress environment. Proliferating tumor cells can also exert solid stresses on the surrounding vasculature, causing blood and lymphatic vessels to collapse and inducing heterogeneous blood flows and subsequent shear stresses (6, 30). Given that fluid shear stress can affect EC glycocalyx thickness and composition, it is likely that alterations are also present in the cancer microenvironment. Such shear-induced effects on the EC glycocalyx have not been previously characterized and could provide new insight into the vascular transport of CTCs.

### Glycocalyx effects on leukocyte adhesion.

Like CTCs, leukocytes express selectin ligands that facilitate their adhesion to the endothelium. Given that selectins on the EC surface do not extend as far from the EC surface as the glycocalyx, it is likely that inflammatory conditions, MMP exposure, and fluid shear forces can remodel the glycocalyx to promote the adhesion of circulating cells. A theoretical model was developed by Zhao et al. (140) to examine how fluid shear forces can amplify the penetration forces of leukocyte microvilli into the EC glycocalyx. Leukocyte microvilli range from 0.3 to 0.7 μm in length, which can place leukocytes within a reactive distance to selectins on the EC surface (10). The model predicts that physiological shear forces can amplify the gravitational contact forces of leukocytes by almost two orders of magnitude to 100 pN, which can allow leukocyte microvilli to penetrate the EC glycocalyx (140).

In vivo, TNF-α was found to disrupt the glycocalyx of postcapillary venules, decrease leukocyte rolling velocity, and increase the number of adherent leukocytes (41). Treatment with the P-selectin antagonist fucoidan reduced the number of adherent leukocytes and increased the average leukocyte rolling velocity, demonstrating that TNF-α-induced glycocalyx disruption enhances selectin-mediated leukocyte adhesion (41). Superperfusion of the rat mesentery with fMLP increased glycocalyx shedding in a process mediated by EC G protein signaling (86), increasing exposure of EC adhesion receptors and subsequent leukocyte adhesion (85). Treatment of mouse cremaster venules with the enzyme heparitinase degraded heparan sulfate from the EC glycocalyx and increased the number of leukocytes adhered to the venules (16). Doxycycline, a tetracycline antibiotic, is a broad-spectrum MMP inhibitor that was recently shown to promote and inhibit leukocyte adhesion (69, 70). Superperfusion of rat mesentery with doxycycline alone increased baseline levels of leukocyte adhesion by reducing sheddase activity and subsequent cleavage of adhesion molecules (70). However, treatment with fMLP followed by doxycycline significantly reduced leukocyte adhesion compared with treatment with fMLP alone, indicating that doxycycline treatment can also inhibit fMLP-induced glycocalyx shedding and reduce the availability of EC adhesion molecules (70).

Under normal physiological conditions, the thick EC glycocalyx can serve as a physical barrier that prevents the adhesion of immune cells and CTCs (Fig. 2*A*). This barrier can be compromised by a variety of factors, including proinflammatory molecules, MMPs, and fluid shear stress. Given the presence of selectin ligands on many CTCs, similar to leukocytes, it is expected that glycocalyx shedding and disruption may exert similar effects on the adhesion of CTCs (Fig. 2*B*). However, CTCs can differ greatly from leukocytes in terms of their size, morphology, selectin ligand expression, deformability, and membrane composition (33, 37, 61, 75, 100, 116, 127, 141). Additionally, CTCs can possess glycocalyx components such as chondroitin sulfate GAGs, which serve as major P-selectin ligands on metastatic breast cancer cells (84). Thus the effects of glycocalyx thickness and composition on CTC adhesion, and subsequent formation of distant metastases, are not fully elucidated and deserve further study.

## Glycocalyx Effects on Therapeutic Treatment of CTCs

### CTC-targeted therapies.

**Fig. 3.**
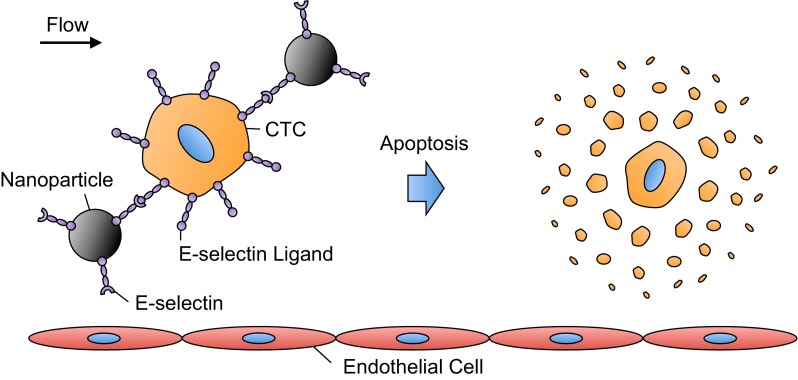
E-selectin-mediated delivery of therapeutics to CTCs in the bloodstream. Under physiological flow conditions, E-selectin-conjugated nanoparticles can rapidly bind to E-selectin ligands on the CTC surface. This adhesive interaction can be utilized to deliver a variety of therapeutic ligands or small molecules to CTCs, which can then induce CTC apoptosis.

Therapeutic treatment of CTCs in blood can potentially hinder the vascular transport of CTCs to anatomically distant organs and prevent the onset of metastasis. Recently, novel approaches have been developed to target and treat CTCs within the bloodstream (56). E-selectin is currently being explored to target therapeutics to CTCs under blood flow conditions, due to the rapid, force-dependent binding kinetics between E-selectin and selectin ligands on CTCs (66) (Fig. 3). Our group recently developed an approach to deliver doxorubicin-containing E-selectin-conjugated nanoparticles to flowing tumor cells (78, 79). Doxorubicin, an adriamyacin anthracycline antibiotic that is utilized as a chemotherapeutic agent, can induce tumor cell death by DNA intercalation, inhibition of topoisomerase II, and formation of free radicals (7, 89, 135). Under physiological shear stresses, tumor cells rapidly bound to E-selectin-conjugated nanoparticles, as confocal microscopy revealed fluorescent nanoparticles decorating the tumor cell surface (79). Doxorubicin-loaded nanoparticles were subsequently internalized by tumor cells and induced significant cell death.

The use of TNF-related apoptosis-inducing ligand (TRAIL) to induce apoptosis in circulating cancer cells has recently been investigated. TRAIL binds to the trimeric death receptors DR4 and DR5 on the surface of a variety of tumor cells and subsequently signals for apoptosis (97). TRAIL is an ideal therapeutic for CTCs, because it does not exert toxic effects on most normal cells, with the exception of hepatocytes (3, 53). Thus upon delivery to the bloodstream, TRAIL would likely have negligible effects on circulating cells such as erythrocytes and leukocytes. Utilizing immobilized E-selectin and TRAIL, Rana et al. (97, 98) created novel surfaces to capture and deliver apoptotic signals to flowing cancer cells. Cancer cells exhibited rolling adhesion on E-selectin and subsequently interacted with immobilized TRAIL on the surface to induce apoptosis (97). Aspirin pretreatment followed by perfusion over E-selectin/TRAIL surfaces sensitized tumor cells to TRAIL-induced apoptosis, significantly increasing the number of apoptotic tumor cells compared with cells exposed to E-selectin/TRAIL surfaces alone (98). Mitchell et al. (81) assessed the effects of physiological shear stress exposure on TRAIL-induced apoptosis of tumor cells. Fluid shear stress exposure of 2.0 dyn/cm^2^ nearly doubled the amount of apoptotic cancer cells in the presence of TRAIL compared with TRAIL-treated cells exposed to static conditions (81). Interestingly, the response was found to be TRAIL-specific, as shear forces did not sensitize cancer cells to doxorubicin-induced apoptosis (81).

### Glycocalyx as a therapeutic barrier.

**Fig. 4.**
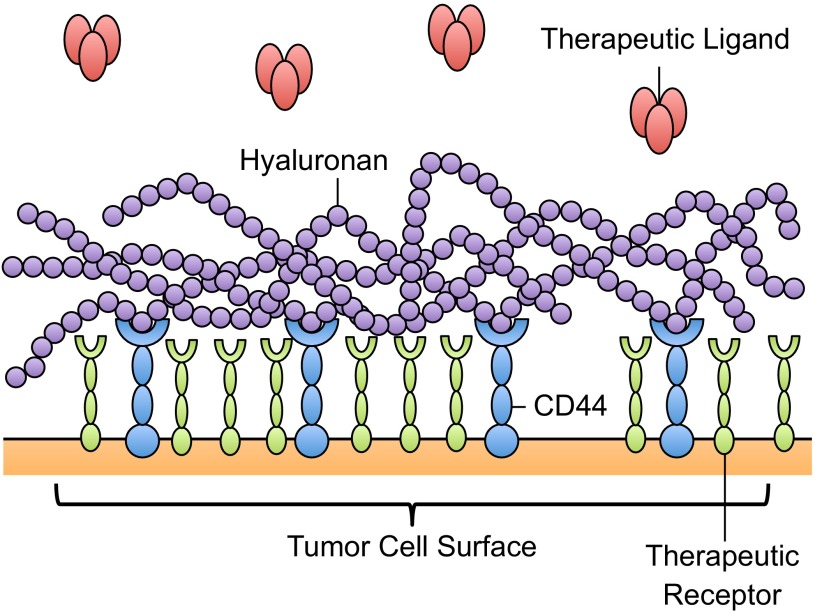
Glycocalyx as a barrier to therapeutic delivery. Overexpression of glycocalyx components, such as hyaluronan, can create a molecular barrier to “shield” therapeutic-binding receptors from interaction with therapeutic ligands.

Increased expression of hyaluronan on the tumor cell glycocalyx can hinder the delivery of therapeutics. High expression levels of hyaluronan can create a hydrated connective tissue matrix, which can attach to the tumor cell surface via CD44 to form a protective coating around the cell (102). This coating may limit therapeutic efficacy by providing a cover over drug binding sites on cancer cells (Fig. 4), along with attenuating the diffusion of drug molecules to the cell surface. Increased production of hyaluronan was found to increase interstitial fluid pressure in solid tumors, which can limit the delivery of therapeutics via the circulation by collapsing nearby blood vessels (6, 30) and eliminating pressure difference-driven transport of therapeutics toward the tumor interior (8). To quantify glycocalyx effects on the diffusion of drugs to tumor cells, Eikenes et al. (25) used fluorescence recovery after photobleaching, based on two-photon scanning laser excitation, to measure the diffusion of FITC-conjugated dextran macromolecules through the glycocalyx. The diffusion coefficient of 150-kDa FITC dextran molecules decreased in tumor spheroids, and even more so in human osteosarcoma xenografts, in part due to the presence of hyaluronan (25). The delivery of drugs such as docetaxel and liposomal doxorubicin to PC-3 tumors was reduced due to the accumulation of hyaluronan (119). Pályi-Krekk et al. (93, 94) investigated the role of hyaluronan and CD44 in trastuzumab resistance. Trastuzumab is a recombinant humanized anti-ErbB2 antibody used in the treatment of breast cancer; however, the mechanisms of resistance are poorly understood (93). CD44 was found to be overexpressed in the trastuzumab-resistant cell line JIMT-1, and flow cytometry fluorescence resonance energy transfer measurements showed that CD44 interacts with ErbB2 (93). In mouse JIMT-1 xenografts, CD44 enhanced trastuzumab internalization, while hyaluronan blocked the availability of ErbB2 to bind to trastuzumab, implicating the CD44-hyaluronan complex in the attenuation of receptor-mediated therapy of tumor cells (93).

### Tumor cell glycocalyx-targeted treatments.

Glycocalyx-degrading enzymes, such as hyaluronidases, have been explored as agents to degrade the glycocalyx coating and subsequently increase therapeutic diffusion and uptake to tumor cells. Early work by Brekken and de Lange Davies (8) investigated the effects of hyaluronidase on interstitial fluid pressure in solid tumors. Intratumoral injections of bovine testicular hyaluronidase reduced interstitial fluid pressure of solid tumors up to 40% at 60 min postinjection (8). Hyaluronidase treatment increased the diffusion of FITC-conjugated dextran macromolecules in human osteosarcomas grown as tumor spheroids and in tumor tissue in vivo, as measured using fluorescence recovery after photobleaching (25). In terms of therapeutic delivery, hyaluronidase-treated orthotopic tumors increased the uptake and distribution of liposomal doxorubicin (27). Thompson et al. (119) utilized the recombinant human hyaluronan-degrading enzyme rHuPH20 to deplete hyaluronan from the tumor cell surface but found that its short serum half-life (<3 min) made in vivo use impractical. However, PEGlyation of rHuPH20 (PEGPH20) increased the serum half-life to >10 h, depleted hyaluronan, decreased interstitial fluid pressure by 84%, and decompressed tumor blood vessels (119). PEGPH20 treatment increased the activity of liposomal doxorubicin and docetaxel in PC-3 tumors.

Given its physiological role and its presence throughout the human body, systemic administration of hyaluronidase could have unwanted side effects. Enzymatic degradation via hyaluronidase can induce inflammation and pain in joints, and because of its pH sensitivity and short half-life in serum, it is difficult to efficiently administer (131). To combat this, Yang et al. (134) recently developed oligosaccharides of hyaluronan (oHA)-lipid-paclitaxel nanoparticles to breach the glycocalyx barrier for therapeutic delivery. oHA is broken down from hyaluronan by hyaluronidase, possesses binding sites for CD44, and can act as an antagonist of CD44 interactions (120, 133). Additionally, because of its small size, oHA nanoparticles are able to breach the glycocalyx barrier to access CD44 receptors. The glycocalyx of MDA-MB-231 and BT-249 breast cancer cells was disrupted via oHA interactions with CD44 and subsequent detachment of hyaluronan from the cell surface (134). Compared with treatment with lipid-paclitaxel nanoparticles alone, which induced apoptosis in ∼30% of both breast cancer cell lines, the addition of oHA to nanoparticles increased apoptosis to >90% of cells (134). The efficacy of the approach was also confirmed in vivo using a mouse xenograft model (134).

Surprisingly, little is known about CTC glycocalyx thickness and composition, and subsequent glycocalyx-mediated CTC resistance to therapeutics. While CTCs in blood are frequently apoptotic (76), it is possible that those that metastasize to anatomically distant organs have distinct survival advantages through retention of their glycocalyx. The glycocalyx can allow CTCs to evade anoikis, while also providing a barrier for therapeutics to bind to cell surface receptors (137). In particular, CTC therapeutic strategies involving E-selectin targeting and TRAIL-induced apoptosis can be affected by the presence of a CTC glycocalyx layer, by blocking interactions with E-selectin ligands and death receptors on CTCs. Glycocalyx-degrading enzymes can thus increase the availability of CTC ligands for therapeutic purposes. In contrast, if CTCs that remain viable do not possess a glycocalyx layer, then targeting mechanisms utilizing E-selectin can provide an efficient method to localize therapeutic ligands to the CTC surface under physiological flow conditions. Future work should focus on direct glycocalyx measurements on CTCs, tumor cell-specific glycocalyx-degrading enzymes to increase the availability of drug binding sites, and subsequent therapeutic treatment of CTCs.

## Conclusion

The transport of CTCs via the bloodstream can be altered due to the presence of the glycocalyx expressed on ECs and CTCs. On the surface of ECs, the glycocalyx can act as a barrier to prevent interactions between adhesion receptors on ECs and ligands on the CTC surface. Inflammation, MMPs, and changes in fluid shear stress can act to disrupt, remodel, and induce shedding of the glycocalyx. These factors can increase the availability of adhesion receptors on the EC surface, which in turn may promote CTC adhesion to the endothelium. Similar adhesive interactions can be utilized to target therapeutics to CTCs, such as apoptosis-inducing ligands, to potentially reduce the spread of metastasis. However, overexpression of hyaluronan leads to the formation of a thicker glycocalyx coating around tumor cells, providing a “shield” against therapeutic ligand delivery to the cell surface. Recent advances that aim to disrupt glycocalyx adhesion to the tumor cell surface provide a means to more effectively deliver therapeutic agents to CTCs. A better understanding of the factors that disrupt the EC glycocalyx to promote tumor cell adhesion, along with strategies to breach CTC therapeutic barriers, will lead to greater control and potential intervention of CTC vascular transport.

## GRANTS

This work was supported by the Cornell Center on the Microenvironment and Metastasis through National Cancer Institute Grant U54 CA143876.

## DISCLOSURES

No conflicts of interest, financial or otherwise, are declared by the authors.

## DISCLAIMER

The content is solely the responsibility of the authors and does not necessarily represent the official views of the National Cancer Institute or the National Institutes of Health.

## AUTHOR CONTRIBUTIONS

M.J.M. prepared the figures; M.J.M. and M.R.K. drafted the manuscript; M.J.M. and M.R.K. edited and revised the manuscript; M.J.M. and M.R.K. approved the final version of the manuscript.

## Footnotes

1 This review is part of a five-article theme series on Physical Biology in Cancer in this issue.

## References

[1] Adamson RH Lenz JF Zhang X Adamson GN Weinbaum S Curry FE Oncotic pressures opposing filtration across non-fenestrated rat microvessels. J Physiol 557: 889–907, 2004 15073281 10.1113/jphysiol.2003.058255 PMC1665140

[2] Arisaka T Mitsumata M Kawasumi M Tohjima T Hirose S Yoshida Y Effects of shear stress on glycosaminoglycan synthesis in vascular endothelial cells. Ann NY Acad Sci 748: 543–554, 1995 7695202 10.1111/j.1749-6632.1994.tb17359.x

[3] Ashkenazi A Targeting death and decoy receptors of the tumour-necrosis factor superfamily. Nat Rev Cancer 2: 420–430, 2002 12189384 10.1038/nrc821

[4] Barthel SR Wiese GK Cho J Opperman MJ Hays DL Siddiqui J Pienta KJ Furie B Dimitroff CJ α_1,3_-Fucosyltransferases are master regulators of prostate cancer cell trafficking. Proc Natl Acad Sci USA 106: 19491–19496, 2009 19889975 10.1073/pnas.0906074106 PMC2780742

[5] Biancone L Araki M Araki K Vassalli P Stamenkovic I Redirection of tumor metastasis by expression of E-selectin in vivo. J Exp Med 183: 581–587, 1996 8627169 10.1084/jem.183.2.581 PMC2192458

[6] Boucher Y Jain RK Microvascular pressure is the principal driving force for interstitial hypertension in solid tumors: implications for vascular collapse. Cancer Res 52: 5110–5114, 1992 1516068

[7] Bouma J Beijnen JH Bult A Underberg WJ Anthracycline antitumour agents. Pharm Weekbl Sci 8: 109–133, 1986 3520474 10.1007/BF02086146

[8] Brekken C de Lange Davies C Hyaluronidase reduces the interstitial fluid pressure in solid tumours in a non-linear concentration-dependent manner. Cancer Lett 131: 65–70, 1998 9839621 10.1016/s0304-3835(98)00202-x

[9] Brodt P Fallavollita L Bresalier RS Meterissian S Norton CR Wolitzky BA Liver endothelial E-selectin mediates carcinoma cell adhesion and promotes liver metastasis. Int J Cancer 71: 612–619, 1997 9178816 10.1002/(sici)1097-0215(19970516)71:4<612::aid-ijc17>3.0.co;2-d

[10] Bruehl RE Springer TA Bainton DF Quantitation of L-selectin distribution on human leukocyte microvilli by immunogold labeling and electron microscopy. J Histochem Cytochem 44: 835–844, 1996 8756756 10.1177/44.8.8756756

[11] Burdick MM Chu JT Godar S Sackstein R HCELL is the major E- and L-selectin ligand expressed on LS174T colon carcinoma cells. J Biol Chem 281: 13899–13905, 2006 16565092 10.1074/jbc.M513617200

[12] Cao TM Mitchell MJ Liesveld J King MR Stem cell enrichment with selectin receptors: mimicking the pH environment of trauma. Sensors 13: 12516–12526, 2013 24048341 10.3390/s130912516 PMC3821329

[13] Chaffer CL Weinberg RA A perspective on cancer cell metastasis. Science 331: 1559–1564, 2011 21436443 10.1126/science.1203543

[14] Chambers AF MacDonald IC Schmidt EE Koop S Morris VL Khokha R Groom AC Steps in tumor metastasis: new concepts from intravital microscopy. Cancer Metastasis Rev 14: 279–301, 1995 8821091 10.1007/BF00690599

[15] Cheung LS Raman PS Balzer EM Wirtz D Konstantopoulos K Biophysics of selectin-ligand interactions in inflammation and cancer. Phys Biol 8: 015013, 2011 21301059 10.1088/1478-3975/8/1/015013

[16] Constantinescu AA Vink H Spaan J Endothelial cell glycocalyx modulates immobilization of leukocytes at the endothelial surface. Arterioscler Thromb Vasc Biol 23: 1541–1547, 2003 12855481 10.1161/01.ATV.0000085630.24353.3D

[17] Constantinescu AA Vink H Spaan JAE Elevated capillary tube hematocrit reflects degradation of endothelial cell glycocalyx by oxidized LDL. Am J Physiol Heart Circ Physiol 280: H1051–H1057, 2001 11179046 10.1152/ajpheart.2001.280.3.H1051

[18] Coussens LM Fingleton B Matrisian LM Matrix metalloproteinase inhibitors and cancer: trials and tribulations. Science 295: 2387–2392, 2002 11923519 10.1126/science.1067100

[19] Coussens LM Werb Z Inflammation and cancer. Nature 420: 860–867, 2002 12490959 10.1038/nature01322 PMC2803035

[20] Damiano ER Long DS El-Khatib FH On the motion of a sphere in a Stokes flow parallel to a Brinkman half-space. J Fluid Mech 500: 75–101, 2004

[21] Damiano ER Long DS Smith ML Estimation of viscosity profiles using velocimetry data from parallel flows of linearly viscous fluids: application to microvascular haemodynamics. J Fluid Mech 512: 1–19, 2004

[22] Desjardins C Duling BR Heparinase treatment suggests a role for the endothelial cell glycocalyx in regulation of capillary hematocrit. Am J Physiol Heart Circ Physiol 258: H647–H654, 1990 10.1152/ajpheart.1990.258.3.H647 2316679

[23] Dimitroff CJ Lechpammer M Long-Woodward D Kutok J Rolling of human bone-metastatic prostate tumor cells on human bone marrow endothelium under shear flow is mediated by E-selectin. Cancer Res 64: 5261–5269, 2004 15289332 10.1158/0008-5472.CAN-04-0691

[24] Duda DG Duyverman AM Kohno M Snuderl M Steller EJ Fukumura D Jain RK Malignant cells facilitate lung metastasis by bringing their own soil. Proc Natl Acad Sci USA 107: 21677–21682, 2010 21098274 10.1073/pnas.1016234107 PMC3003109

[25] Eikenes L Tufto I Schnell EA Bjorkoy A de Lange Davies C Effect of collagenase and hyaluronidase on free and anomalous diffusion in multicellular spheroids and xenografts. Anticancer Res 30: 359–368, 2010 20332440

[26] Endo K Takino T Miyamori H Kinsen H Yoshizaki T Furukawa M Sato H Cleavage of syndecan-1 by membrane type matrix metalloproteinase-1 stimulates cell migration. J Biol Chem 278: 40764–40770, 2003 12904296 10.1074/jbc.M306736200

[27] Erikson A Tufto I Bjønnum AB Bruland ØS de Lange Davies C The impact of enzymatic degradation on the uptake of differently sized therapeutic molecules. Anticancer Res 28: 3557–3566, 2008 19189635

[28] Fidler I The pathogenesis of cancer metastasis: the “seed and soil” hypothesis revisited. Nat Rev Cancer 3: 1–6, 2003 10.1038/nrc1098 12778135

[29] Fitzgerald ML Wang Z Park PW Murphy G Bernfield M Shedding of syndecan-1 and -4 ectodomains is regulated by multiple signaling pathways and mediated by a TIMP-3-sensitive metalloproteinase. J Cell Biol 148: 811–824, 2000 10684261 10.1083/jcb.148.4.811 PMC2169376

[30] Fukumura D Jain RK Tumor microenvironment abnormalities: causes, consequences, and strategies to normalize. J Cell Biochem 101: 937–949, 2007 17171643 10.1002/jcb.21187

[31] Galis ZS Sukhova GK Lark MW Libby P Increased expression of matrix metalloproteinases and matrix degrading activity in vulnerable regions of human atherosclerotic plaques. J Clin Invest 94: 2493–2503, 1994 7989608 10.1172/JCI117619 PMC330083

[32] Giavazzi R Foppolo M Dossi R Remuzzi A Rolling and adhesion of human tumor cells on vascular endothelium under physiological flow conditions. J Clin Invest 92: 3038–3044, 1993 7504697 10.1172/JCI116928 PMC288509

[33] Gout S Tremblay P Huot J Selectins and selectin ligands in extravasation of cancer cells and organ selectivity of metastasis. Clin Exp Metastasis 25: 335–344, 2008 17891461 10.1007/s10585-007-9096-4

[34] Götte M Yip GW Heparanase, hyaluronan, and CD44 in cancers: a breast carcinoma perspective. Cancer Res 66: 10233–10237, 2006 17079438 10.1158/0008-5472.CAN-06-1464

[34a] Grimm J Keller R deGroot PG Laminar flow induces cell polarity and leads to rearrangement of proteoglycan metabolism in endothelial cells. Thromb Haemost 60: 437–441, 1988 3238646

[35] Grivennikov S Greten F Karin M Immunity, inflammation, cancer. Cell 140: 883–899, 2010 20303878 10.1016/j.cell.2010.01.025 PMC2866629

[36] Gronski TJ Martin RL Kobayashi DK Walsh BC Holman MC Huber M Van Wart HE Shapiro SD Hydrolysis of a broad spectrum of extracellular matrix proteins by human macrophage elastase. J Biol Chem 272: 12189–12194, 1997 9115292 10.1074/jbc.272.18.12189

[37] Guck J Schinkinger S Lincoln B Wottawah F Ebert S Romeyke M Lenz D Erickson HM Ananthakrishnan R Mitchell D Käs J Ulvick S Bilby C Optical deformability as an inherent cell marker for testing malignant transformation and metastatic competence. Biophys J 88: 3689–3698, 2005 15722433 10.1529/biophysj.104.045476 PMC1305515

[38] Günthert U Hofmann M Rudy W Reber S Zöller M Haussmann I Matzku S Wenzel A Ponta H Herrlich P A new variant of glycoprotein CD44 confers metastatic potential to rat carcinoma cells. Cell 65: 13–24, 1991 1707342 10.1016/0092-8674(91)90403-l

[39] Hamerman D Todaro GJ Green H The production of hyaluronate by spontaneously established cell lines and viral transformed lines of fibroblastic origin. Biochim Biophys Acta 101: 343–351, 1965 4285883 10.1016/0926-6534(65)90013-8

[40] Henry CB Duling BR Permeation of the luminal capillary glycocalyx is determined by hyaluronan. Am J Physiol Heart Circ Physiol 277: H508–H514, 1999 10.1152/ajpheart.1999.277.2.H508 10444475

[41] Henry CB Duling BR TNF-α increases entry of macromolecules into luminal endothelial cell glycocalyx. Am J Physiol Heart Circ Physiol 279: H2815–H2823, 2000 11087236 10.1152/ajpheart.2000.279.6.H2815

[42] Hofmann-Kiefer KF Kemming GI Chappell D Flondor M Kisch-Wedel H Hauser A Pallivathukal S Conzen P Rehm M Serum heparan sulfate levels are elevated in endotoxemia. Eur J Med Res 14: 526–531, 2009 20149986 10.1186/2047-783X-14-12-526 PMC3351938

[43] Hopwood JJ Dorfman A Glycosaminoglycan synthesis by cultured human skin fibroblasts after transformation with simian virus 40. J Biol Chem 252: 4777–4785, 1977 194893

[44] Hou JM Krebs MG Lancashire L Sloane R Backen A Swain RK Priest LJ Greystoke A Zhou C Morris K Ward T Blackhall FH Dive C Clinical significance and molecular characteristics of circulating tumor cells and circulating tumor microemboli in patients with small-cell lung cancer. J Clin Oncol 30: 525–532, 2012 22253462 10.1200/JCO.2010.33.3716

[45] Hu X Weinbaum S A new view of Starling's hypothesis at the microstructural level. Microvasc Res 58: 281–304, 1999 10527770 10.1006/mvre.1999.2177

[46] Hughes B Hollers J Crockett-Torabi E Smith CW Recruitment of CD11b/CD18 to the neutrophil surface and adherence-dependent cell locomotion. J Clin Invest 90: 1687–1696, 1992 1358917 10.1172/JCI116041 PMC443225

[47] Huxley VH Curry FE Differential actions of albumin and plasma on capillary solute permeability. Am J Physiol Heart Circ Physiol 260: H1645–H1654, 1991 10.1152/ajpheart.1991.260.5.H1645 2035684

[48] Itano N Kimata K Altered hyaluronan biosynthesis in cancer progression. Semin Cancer Biol 18: 268–274, 2008 18450474 10.1016/j.semcancer.2008.03.006

[49] Itano N Sawai T Miyaishi O Kimata K Relationship between hyaluronan production and metastatic potential of mouse mammary carcinoma cells. Cancer Res 59: 2499–2504, 1999 10344764

[50] Itano N Sawai T Yoshida M Lenas P Yamada Y Imagawa M Shinomura T Hamaguchi M Yoshida Y Ohnuki Y Miyauchi S Spicer AP McDonald JA Kimata K Three isoforms of mammalian hyaluronan synthases have distinct enzymatic properties. J Biol Chem 274: 25085–25092, 1999 10455188 10.1074/jbc.274.35.25085

[51] Jackson RL Busch SJ Cardin AD Glycosaminoglycans: molecular properties, protein interactions, and role in physiological processes. Physiol Rev 71: 481–539, 1991 2006221 10.1152/physrev.1991.71.2.481

[52] Jain RK Determinants of tumor blood flow: a review. Cancer Res 48: 2641–2658, 1988 3282647

[53] Jo M Kim TH Seol DW Esplen JE Dorko K Billiar TR Strom SC Apoptosis induced in normal human hepatocytes by tumor necrosis factor-related apoptosis-inducing ligand. Nat Med 6: 564–567, 2000 10802713 10.1038/75045

[54] Kalluri R Weinberg R The basics of epithelial-mesenchymal transition. J Clin Invest 119: 1420, 2009 19487818 10.1172/JCI39104 PMC2689101

[55] Keller MW Damon DN Duling BR Determination of capillary tube hematocrit during arteriolar microperfusion. Am J Physiol Heart Circ Physiol 266: H2229–H2238, 1994 10.1152/ajpheart.1994.266.6.H2229 7517645

[56] King MR Rolling in the deep: therapeutic targeting of circulating tumor cells. Front Oncol 2: 184, 2012 23226682 10.3389/fonc.2012.00184 PMC3509341

[57] Klingbeil P Natrajan R Everitt G Vatcheva R Marchio C Palacios J Buerger R Reis-Filho JS Isacke CM CD44 is overexpressed in basal-like breast cancers but is not a driver of 11p13 amplification. Breast Cancer Res Treat 120: 95–109, 2010 19350388 10.1007/s10549-009-0380-7

[58] Klitzman B Duling BR Microvascular hematocrit and red cell flow in resting and contracting striated muscle. Am J Physiol Heart Circ Physiol 237: H481–H490, 1979 10.1152/ajpheart.1979.237.4.H481 495734

[59] Koo A Dewey CF Garcia-Cardena G Hemodynamic shear stress characteristic of atherosclerosis-resistant regions promotes glycocalyx formation in cultured endothelial cells. Am J Physiol Cell Physiol 304: C137–C146, 2013 23114962 10.1152/ajpcell.00187.2012 PMC3546807

[60] Kosaki R Watanabe K Yamaguchi Y Overproduction of hyaluronan by expression of the hyaluronan synthase Has2 enhances anchorage-independent growth and tumorigenicity. Cancer Res 59: 1141–1145, 1999 10070975

[61] Kuo JS Zhao Y Schiro PG Ng L Lim DS Shelby JP Chiu DT Deformability considerations in filtration of biological cells. Lab Chip 10: 837–842, 2010 20379567 10.1039/b922301k

[62] Lawrence MB Springer TA Leukocytes roll on a selectin at physiologic flow rates: distinction from and prerequisite for adhesion through integrins. Cell 65: 859–873, 1991 1710173 10.1016/0092-8674(91)90393-d

[63] Lawrence MB Springer TA Neutrophils roll on E-selectin. J Immunol 151: 6339–6346, 1993 7504018

[64] Lee D Schultz JB Knauf PA King MR Mechanical shedding of L-selectin from the neutrophil surface during rolling on sialyl Lewis X under flow. J Biol Chem 282: 4812–4820, 2007 17172469 10.1074/jbc.M609994200

[65] Leonard JG Hale AH Roll DE Conrad HE Weber MJ Turnover of cellular carbohydrates in normal and Rous sarcoma virus-transformed cells. Cancer Res 38: 185–188, 1978 201373

[66] Li J King MR Adhesion receptors as therapeutic targets for circulating tumor cells. Front Oncol 2: 1–9, 2012 10.3389/fonc.2012.00079 PMC3402858 22837985

[67] Li Y Heldin P Hyaluronan production increases the malignant properties of mesothelioma cells. Br J Cancer 85: 600–607, 2001 11506502 10.1054/bjoc.2001.1922 PMC2364109

[68] Li Z Li L Zielke R Cheng L Xiao R Crow MT Stetler-Stevenson WG Froehlich J Lakatta EG Increased expression of 72-kd type IV collagenase (MMP-2) in human aortic atherosclerotic lesions. Am J Pathol 148: 121, 1996 8546199 PMC1861591

[69] Lipowsky HH Lescanic A The effect of doxycycline on shedding of the glycocalyx due to reactive oxygen species. *Microvasc Res*. In press. 10.1016/j.mvr.2013.07.004 PMC3852187 23899417

[70] Lipowsky HH Sah R Lescanic A Relative roles of doxycycline and cation chelation in endothelial glycan shedding and adhesion of leukocytes. Am J Physiol Heart Circ Physiol 300: H415–H422, 2011 21148759 10.1152/ajpheart.00923.2010 PMC3044056

[71] Lipowsky HH The endothelial glycocalyx as a barrier to leukocyte adhesion and its mediation by extracellular proteases. Ann Biomed Eng 40: 840–848, 2012 21984514 10.1007/s10439-011-0427-x PMC3306510

[72] Liu N Gao F Han Z Xu X Underhill CB Zhang L Hyaluronan synthase 3 overexpression promotes the growth of TSU prostate cancer cells. Cancer Res 61: 5207–5214, 2001 11431361

[73] Madlener M Parks WC Werner S Matrix metalloproteinases (MMPs) and their physiological inhibitors (TIMPs) are differentially expressed during excisional skin wound repair. Exp Cell Res 242: 201–210, 1998 9665817 10.1006/excr.1998.4049

[74] Martin P Wound healing—aiming for perfect skin regeneration. Science 276: 75–81, 1997 9082989 10.1126/science.276.5309.75

[75] Matsumoto S Imaeda Y Umemoto S Kobayashi K Suzuki H Okamoto T Cimetidine increases survival of colorectal cancer patients with high levels of sialyl Lewis-X and sialyl Lewis-A epitope expression on tumour cells. Br J Cancer 86: 161–167, 2002 11870500 10.1038/sj.bjc.6600048 PMC2375187

[76] Méhes G Witt A Kubista E Ambros PF Circulating breast cancer cells are frequently apoptotic. Am J Pathol 159: 17–20, 2001 11438448 10.1016/S0002-9440(10)61667-7 PMC1850424

[77] Michel CC Starling: the formulation of his hypothesis of microvascular fluid exchange and its significance after 100 years. Exp Physiol 82: 1–30, 1997 9023503 10.1113/expphysiol.1997.sp004000

[78] Mitchell MJ Castellanos CA King MR Nanostructured surfaces to target and kill circulating tumor cells while repelling leukocytes. J Nanomaterials 2012: 1–10, 2012 10.1155/2012/831263 PMC4139011 25152752

[79] Mitchell MJ Chen CS Ponmudi V Hughes AD King MR E-selectin liposomal and nanotube-targeted delivery of doxorubicin to circulating tumor cells. J Control Release 160: 609–617, 2012 22421423 10.1016/j.jconrel.2012.02.018 PMC3749772

[80] Mitchell MJ King MR Shear-induced resistance to neutrophil activation via the formyl peptide receptor. Biophys J 102: 1804–1814, 2012 22768936 10.1016/j.bpj.2012.03.053 PMC3328724

[81] Mitchell MJ King MR Fluid shear stress sensitizes cancer cells to receptor-mediated apoptosis via trimeric death receptors. New J Physics 15: 015008, 2013 10.1088/1367-2630/15/1/015008 PMC4124740 25110459

[82] Mitchell MJ King MR Computational and experimental models of cancer cell response to fluid shear stress. Front Oncol 3: 1–11, 2013 23467856 10.3389/fonc.2013.00044 PMC3587800

[83] Mitsiades N Yu W Poulaki V Tsokos M Stamenkovic I Matrix metalloproteinase-7-mediated cleavage of Fas ligand protects tumor cells from chemotherapeutic drug cytotoxicity. Cancer Res 61: 577–581, 2001 11212252

[84] Monzavi-Karbassi B Stanley JS Hennings L Jousheghany F Artaud C Shaaf S Kieber-Emmons T Chondroitin sulfate glycosaminoglycans as major P-selectin ligands on metastatic breast cancer cell lines. Int J Cancer 120: 1179–1191, 2007 17154173 10.1002/ijc.22424

[85] Mulivor AW Lipowsky HH Role of glycocalyx in leukocyte-endothelial cell adhesion. Am J Physiol Heart Circ Physiol 283: H1282–H1291, 2002 12234777 10.1152/ajpheart.00117.2002

[86] Mulivor AW Lipowsky HH Inflammation- and ischemia-induced shedding of venular glycocalyx. Am J Physiol Heart Circ Physiol 286: H1672–H1680, 2004 14704229 10.1152/ajpheart.00832.2003

[87] Nagano O Okazaki S Saya H Redox regulation in stem-like cancer cells by CD44 variant isoforms. Oncogene 32: 5191–5198, 2013 23334333 10.1038/onc.2012.638

[88] Oohira A Wight TN Bornstein P Sulfated proteoglycans synthesized by vascular endothelial cells in culture. J Biol Chem 258: 2014–2021, 1983 6337150

[89] Osheroff N Corbett AH Robinson MJ Mechanism of action of topoisomerase II-targeted antineoplastic drugs. Adv Pharmacol 29: 105–126, 1994 8996604 10.1016/s1054-3589(08)61134-5

[90] Pang Z Tarbell JM In vitro study of Starling's hypothesis in a cultured monolayer of bovine aortic endothelial cells. J Vasc Res 40: 351–358, 2003 12891004 10.1159/000072699

[91] Paszek MJ DuFort CC Rubashkin MG Davidson MW Thorn KS Liphardt JT Weaver VM Scanning angle interference microscopy reveals cell dynamics at the nanoscale. Nat Methods 9: 825–827, 2012 22751201 10.1038/nmeth.2077 PMC3454456

[92] Paterlini-Brechot P Benali NL Circulating tumor cells (CTC) detection: clinical impact and future directions. Cancer Lett 253: 180–204, 2007 17314005 10.1016/j.canlet.2006.12.014

[93] Pályi-Krekk Z Barok M Isola J Tammi M Szöllosi J Nagy P Hyaluronan-induced masking of ErbB2 and CD44-enhanced trastuzumab internalisation in trastuzumab resistant breast cancer. Eur J Cancer 43: 2423–2433, 2007 17911008 10.1016/j.ejca.2007.08.018

[94] Pályi-Krekk Z Barok M Kovács T Saya H Nagano O Szöllosi J Nagy P EGFR and ErbB2 are functionally coupled to CD44 and regulate shedding, internalization and motogenic effect of CD44. Cancer Lett 263: 231–242, 2008 18276068 10.1016/j.canlet.2008.01.014

[95] Pries AR Secomb TW Gaehtgens P The endothelial surface layer. Pflügers Arch 440: 653–666, 2000 11007304 10.1007/s004240000307

[96] Prince ME Sivanandan R Kaczorowski A Wolf GT Kaplan MJ Dalerba P Weissman IL Clarke MF Ailles LE Identification of a subpopulation of cells with cancer stem cell properties in head and neck squamous cell carcinoma. Proc Natl Acad Sci USA 104: 973–978, 2007 17210912 10.1073/pnas.0610117104 PMC1783424

[97] Rana K Liesveld JL King MR Delivery of apoptotic signal to rolling cancer cells: a novel biomimetic technique using immobilized TRAIL and E-selectin. Biotechnol Bioeng 102: 1692–1702, 2009 19073014 10.1002/bit.22204

[98] Rana K Reinhart-King CA King MR Inducing apoptosis in rolling cancer cells: a combined therapy with aspirin and immobilized TRAIL and E-selectin. Mol Pharm 9: 2219–2227, 2012 22724630 10.1021/mp300073j PMC3412427

[99] Rehm M Bruegger D Christ F Conzen P Thiel M Jacob M Chappell D Stoeckelhuber M Welsch U Reichart B Peter K Becker BF Shedding of the endothelial glycocalyx in patients undergoing major vascular surgery with global and regional ischemia. Circulation 116: 1896–1906, 2007 17923576 10.1161/CIRCULATIONAHA.106.684852

[100] Reis CA Osorio H Silva L Gomes C David L Alterations in glycosylation as biomarkers for cancer detection. J Clin Pathol 63: 322–329, 2010 20354203 10.1136/jcp.2009.071035

[101] Reitsma S Slaaf DW Vink H Zandvoort MA oude Egbrink MG The endothelial glycocalyx: composition, functions, and visualization. Pflügers Arch 454: 345–359, 2007 17256154 10.1007/s00424-007-0212-8 PMC1915585

[102] Rilla K Tiihonen R Kultti A Tammi M Tammi R Pericellular hyaluronan coat visualized in live cells with a fluorescent probe is scaffolded by plasma membrane protrusions. J Histochem Cytochem 56: 901–910, 2008 18574248 10.1369/jhc.2008.951665 PMC2544615

[103] Robert P Nicolas A Aranda-Espinoza S Bongrand P Limozin L Minimal encounter time and separation determine ligand-receptor binding in cell adhesion. Biophys J 100: 2642–2651, 2011 21641309 10.1016/j.bpj.2011.04.011 PMC3117183

[104] Rosenberg RD Shworak NW Liu J Schwartz JJ Zhang L Heparan sulfate proteoglycans of the cardiovascular system. Specific structures emerge but how is synthesis regulated? J Clin Invest 99: 2062–2070, 1997 9151776 10.1172/JCI119377 PMC508034

[105] Rostgaard J Qvortrup K Electron microscopic demonstrations of filamentous molecular sieve plugs in capillary fenestrae. Microvasc Res 53: 1–13, 1997 9056471 10.1006/mvre.1996.1987

[106] Sabri S Soler M Foa C Pierres A Benoliel A Bongrand P Glycocalyx modulation is a physiological means of regulating cell adhesion. J Cell Sci 113: 1589–1600, 2000 10751150 10.1242/jcs.113.9.1589

[107] Sarelius IH Duling BR Direct measurement of microvessel hematocrit, red cell flux, velocity, and transit time. Am J Physiol Heart Circ Physiol 243: H1018–H1026, 1982 10.1152/ajpheart.1982.243.6.H1018 7149038

[108] Seiter S Arch R Reber S Komitowski D Hofmann M Ponta H Herrlich P Matzku S Zöller M Prevention of tumor metastasis formation by anti-variant CD44. J Exp Med 177: 443–455, 1993 8426113 10.1084/jem.177.2.443 PMC2190906

[109] Sironen RK Tammi M Tammi R Auvinen PK Anttila M Kosma VM Hyaluronan in human malignancies. Exp Cell Res 317: 383–391, 2011 21134368 10.1016/j.yexcr.2010.11.017

[110] Soler M Desplat-Jego S Vacher B Ponsonnet L Fraterno M Bongrand P Martin JM Foa C Adhesion-related glycocalyx study: quantitative approach with imaging-spectrum in the energy filtering transmission electron microscope (EFTEM). FEBS Lett 429: 89–94, 1998 9657389 10.1016/s0014-5793(98)00570-5

[111] Soler M Merant C Servant C Fraterno M Allasia C Lissitzky JC Bongrand P Foa C Leukosialin (CD43) behavior during adhesion of human monocytic THP-1 cells to red blood cells. J Leukoc Biol 61: 609–618, 1997 9129210 10.1002/jlb.61.5.609

[112] Springer TA Adhesion receptors of the immune system. Nature 346: 425–434, 1990 1974032 10.1038/346425a0

[113] Springer TA Traffic signals for lymphocyte recirculation and leukocyte emigration: the multistep paradigm. Cell 76: 301–314, 1994 7507411 10.1016/0092-8674(94)90337-9

[114] Squire JM Chew M Nneji G Neal C Barry J Michel C Quasi-periodic substructure in the microvessel endothelial glycocalyx: a possible explanation for molecular filtering? J Struct Biol 136: 239–255, 2001 12051903 10.1006/jsbi.2002.4441

[115] Swartz MA Lund AW Lymphatic and interstitial flow in the tumour microenvironment: linking mechanobiology with immunity. Nat Rev Cancer 12: 210–219, 2012 22362216 10.1038/nrc3186

[116] Tan SJ Yobas L Lee GY Ong CN Lim CT Microdevice for the isolation and enumeration of cancer cells from blood. Biomed Microdevices 11: 883–892, 2009 19387837 10.1007/s10544-009-9305-9

[117] Taraboletti G D'Ascenzo S Borsotti P Giavazzi R Pavan A Dolo V Shedding of the matrix metalloproteinases MMP-2, MMP-9, and MT1-MMP as membrane vesicle-associated components by endothelial cells. Am J Pathol 160: 673–680, 2002 11839588 10.1016/S0002-9440(10)64887-0 PMC1850663

[118] Thiery JP Epithelial-mesenchymal transitions in tumour progression. Nat Rev Cancer 2: 442–454, 2002 12189386 10.1038/nrc822

[119] Thompson CB Shepard HM O'Connor PM Kadhim S Jiang P Osgood RJ Bookbinder LH Li X Sugarman BJ Connor RJ Nadjsombati S Frost GI Enzymatic depletion of tumor hyaluronan induces antitumor responses in preclinical animal models. Mol Cancer Ther 9: 3052–3064, 2010 20978165 10.1158/1535-7163.MCT-10-0470

[120] Toole BP Slomiany MG Hyaluronan: a constitutive regulator of chemoresistance and malignancy in cancer cells. Semin Cancer Biol 18: 244–250, 2008 18534864 10.1016/j.semcancer.2008.03.009 PMC2517221

[121] Toole BP Wight TN Tammi MI Hyaluronan-cell interactions in cancer and vascular disease. J Biol Chem 277: 4593–4596, 2002 11717318 10.1074/jbc.R100039200

[122] Tözeren A Kleinman HK Grant DS Morales D Mercurio A Byers S E-selectin-mediated dynamic interactions of breast and colon cancer cells with endothelial cell monolayers. Int J Cancer 60: 426–431, 1995 7530236 10.1002/ijc.2910600326

[123] Turitto VT Blood viscosity, mass transport, thrombogenesis. Prog Hemost Thromb 6: 139–177, 1982 6762611

[124] van den Berg BM Vink H Spaan JA The endothelial glycocalyx protects against myocardial edema. Circ Res 92: 592–594, 2003 12637366 10.1161/01.RES.0000065917.53950.75

[125] Vink H Duling BR Identification of distinct luminal domains for macromolecules, erythrocytes, and leukocytes within mammalian capillaries. Circ Res 79: 581–589, 1996 8781491 10.1161/01.res.79.3.581

[126] Vitte J Benoliel AM Pierres A Bongrand P Regulation of cell adhesion. Clin Hemorheol Microcirc 33: 167–188, 2005 16215283

[127] Vona G Sabile A Louha M Sitruk V Romana S Schütze K Capron F Franco D Pazzagli M Vekemans M Lacour B Bréchot C Paterlini-Brechot P Isolation by size of epithelial tumor cells: a new method for the immunomorphological and molecular characterization of circulating tumor cells. Am J Pathol 156: 57–63, 2000 10623654 10.1016/S0002-9440(10)64706-2 PMC1868645

[128] Weigelt B Peterse JL van't Veer LJ Breast cancer metastasis: markers and models. Nat Rev Cancer 5: 591–602, 2005 16056258 10.1038/nrc1670

[129] Weinbaum S Tarbell JM Damiano ER The structure and function of the endothelial glycocalyx layer. Annu Rev Biomed Eng 9: 121–167, 2007 17373886 10.1146/annurev.bioeng.9.060906.151959

[130] Weinbaum S Zhang X Han Y Vink H Cowin SC Mechanotransduction and flow across the endothelial glycocalyx. Proc Natl Acad Sci USA 100: 7988–7995, 2003 12810946 10.1073/pnas.1332808100 PMC164700

[131] Whatcott CJ Han H Posner RG Hostetter G Hoff Von DD Targeting the tumor microenvironment in cancer: why hyaluronidase deserves a second look. Cancer Discovery 1: 291–296, 2011 22053288 10.1158/2159-8290.CD-11-0136 PMC3204883

[132] Wong SY Hynes RO Lymphatic or hematogenous dissemination: how does a metastatic tumor cell decide? Cell Cycle 5: 812–817, 2006 16627996 10.4161/cc.5.8.2646 PMC1459485

[133] Yang C Cao M Liu H He Y Xu J Du Y Liu Y Wang W Cui L Hu J Gao F The high and low molecular weight forms of hyaluronan have distinct effects on CD44 clustering. J Biol Chem 287: 43094–43107, 2012 23118219 10.1074/jbc.M112.349209 PMC3522304

[134] Yang C Liu Y He Y Du Y Wang W Shi X Gao F The use of HA oligosaccharide-loaded nanoparticles to breach the endogenous hyaluronan glycocalyx for breast cancer therapy. Biomaterials 34: 6829–6838, 2013 23764114 10.1016/j.biomaterials.2013.05.036

[135] Young RC Ozols RF Myers CE The anthracycline antineoplastic drugs. N Engl J Med 305: 139–153, 1981 7017406 10.1056/NEJM198107163050305

[136] Yu M Bardia A Wittner BS Stott SL Smas ME Ting DT Isakoff SJ Ciciliano JC Wells MN Shah AM Concannon KF Donaldson MC Sequist LV Brachtel E Sgroi D Baselga J Ramaswamy S Toner M Haber DA Maheswaran S Circulating breast tumor cells exhibit dynamic changes in epithelial and mesenchymal composition. Science 339: 580–584, 2013 23372014 10.1126/science.1228522 PMC3760262

[137] Yu M Stott S Toner M Maheswaran S Haber DA Circulating tumor cells: approaches to isolation and characterization. J Cell Biol 192: 373–382, 2011 21300848 10.1083/jcb.201010021 PMC3101098

[138] Yu WH Woessner JF Heparan sulfate proteoglycans as extracellular docking molecules for matrilysin (matrix metalloproteinase 7). J Biol Chem 275: 4183–4191, 2000 10660581 10.1074/jbc.275.6.4183

[139] Zeng Y Waters M Andrews A Honarmandi P Ebong E Rizzo V Tarbell JM Fluid shear stress induces the clustering of heparan sulfate via mobility of glypican-1 in lipid rafts. Am J Physiol Heart Circ Physiol 305: H811–H820, 2013 23851278 10.1152/ajpheart.00764.2012 PMC3761343

[140] Zhao Y Chien S Weinbaum S Dynamic contact forces on leukocyte microvilli and their penetration of the endothelial glycocalyx. Biophys J 80: 1124–1140, 2001 11222278 10.1016/S0006-3495(01)76090-0 PMC1301309

[141] Zheng S Lin H Liu JQ Balic M Datar R Cote RJ Tai YC Membrane microfilter device for selective capture, electrolysis and genomic analysis of human circulating tumor cells. J Chromatogr A 1162: 154–161, 2007 17561026 10.1016/j.chroma.2007.05.064

